# Non-Lithographic Silicon Micromachining Using Inkjet and Chemical Etching

**DOI:** 10.3390/mi7120222

**Published:** 2016-12-08

**Authors:** Sasha Hoshian, Cristina Gaspar, Teemu Vasara, Farzin Jahangiri, Ville Jokinen, Sami Franssila

**Affiliations:** Department of Chemistry and Materials Science, School of Chemical Technology, Aalto University, FI02150 Espoo, Finland; cristina.henriques.gaspar@aalto.fi (C.G.); teemu.vasara@aalto.fi (T.V.); farzin.jahangiri@aalto.fi (F.J.); ville.p.jokinen@aalto.fi (V.J.)

**Keywords:** non-lithographic, patterning, silicon, micromachining, microfluidic

## Abstract

We introduce a non-lithographical and vacuum-free method to pattern silicon. The method combines inkjet printing and metal assisted chemical etching (MaCE); we call this method “INKMAC”. A commercial silver ink is printed on top of a silicon surface to create the catalytic patterns for MaCE. The MaCE process leaves behind a set of silicon nanowires in the shape of the inkjet printed micrometer scale pattern. We further show how a potassium hydroxide (KOH) wet etching process can be used to rapidly etch away the nanowires, producing fully opened cavities and channels in the shape of the original printed pattern. We show how the printed lines (width 50–100 µm) can be etched into functional silicon microfluidic channels with different depths (10–40 µm) with aspect ratios close to one. We also used individual droplets (minimum diameter 30 µm) to produce cavities with a depth of 60 µm and an aspect ratio of two. Further, we discuss using the structured silicon substrate as a template for polymer replication to produce superhydrophobic surfaces.

## 1. Introduction

Silicon microfabrication techniques are enablers for microelectronics, microelectromechanical systems (MEMS) and microfluidics. The standard cleanroom based fabrication methods are very precise but they are also costly and require significant infrastructure and expertise. In addition to lithography and etching, silicon can be patterned by laser ablation [1,2], ion beam milling [3] and traditional micromachining, such as electro-discharge machining, micro-dicing and cutting [4]. These techniques are limited either by expensive instruments and small writable areas, or by poor resolution and limited shape freedom, which limit their usability. There is thus a need for a rapid prototyping technique that allows fast and simple fabrication of silicon structures without cleanroom facilities. We show that a combination of inkjet printing and wet etching can be utilized to micropattern silicon in the 30 µm to 100 µm size scales. This combination can make anisotropic etching to produce high aspect ratio silicon structures, albeit not at the level of state-of-the-art microfabrication technologies, e.g., deep reactive ion etching and electrochemical micromachining [5,6]. However, these size scales are suitable for applications in microfluidics which we demonstrate by showing fabrication of microfluidic channels in silicon, and superhydrophobic surfaces by polymer replication from silicon. 

Non-lithographic fabrication for microfluidic devices can be done by replica molding using laser-printed or 3D printed masters [7,8,9,10]. The former is limited by height/depth (e.g., 6 µm in a single print step) [11] and the latter by lateral resolution, e.g., 300–400 µm in lateral and 50 µm vertical dimensions (aspect ratios 1:6 only) [12,13]. However, printing also has several notable advantages. Inkjet printing enables cost efficient mass manufacturing of electrodes and other functional materials on large substrates, such as plastic [14], paper [15], fabrics [16] or silicon [17] with a broad area of application. Elimination of lithography means that major equipment become redundant (mask aligner, photoresist spinner) and chemicals like photoresist and developer are not needed.

Metal-assisted chemical etching (MaCE) has attracted attention for both micro and nanostructuring of the silicon substrate [18,19]. It is a simple wet process with the possibility to control different parameters (e.g., cross-sectional shape, diameter and length). The entire process can be done in a chemical lab without expensive equipment. There is also no obvious limitation for the size of the features fabricated by MaCE. In MaCE, an aqueous solution containing hydrogen fluoride (HF) and an oxidant hydrogen peroxide (H_2_O_2_) etches silicon anisotropically when a thin layer of noble metal (e.g., Ag, Au, Pt) is used as a catalyst. Noble metal acts as a cathode that is reduced in the solution and produces holes. These holes diffuse through the noble metal into silicon that is in contact with metal. Silicon is oxidized and the oxide is dissolved by HF, forming nanopillars or pores. Typically, the patterns are defined using various lithographical methods, e.g., photo [19], interface [20] and also colloidal (nanosphere) lithography [21]. Patterning is followed by deposition of noble metals which can be deposited on the Si substrate via various methods, which include thermal evaporation [22], sputtering [23], electron beam (e-beam) evaporation [24], electroless deposition [25], focused-ion-beam (FIB)-assisted deposition [26], or spin-coating of particles via other methods [27].

**Figure 1:**
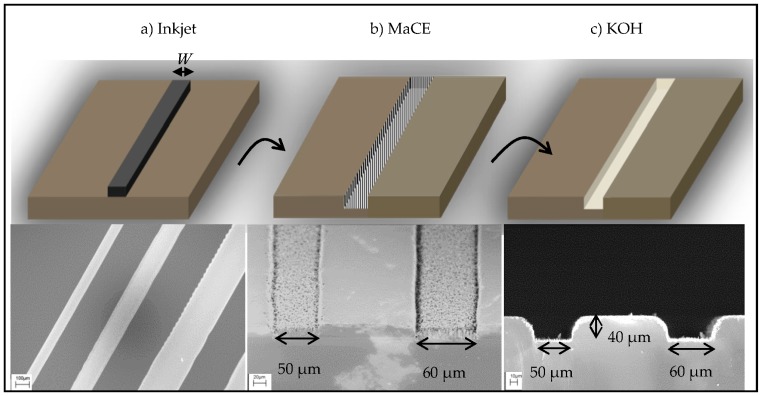
Process flow with corresponding SEM images, (**a**) Inkjetting silver nano-particles; (**b**) Metal assisted chemical etching (MaCE) process to produce silicon nano-wires; (**c**) Potassium hydroxide (KOH) etching of silicon nano-wires to open the channels. “*W*” is the width of channel and ranged from 50 to 100 µm.

In this work, we introduce a non-lithographic and vacuum-free hybrid technique that is a combination of inkjet printing and chemical etching (dubbed INKMAC) to produce cavities and trenches in silicon substrates. The patterns of cavities and trenches are defined with inkjetting of metallic nanoparticles on silicon substrate as dots and lines respectively, followed by chemical etching. The chemical etching part consists of two steps; 1: MaCE to transfer the printed pattern into silicon substrate; 2: potassium hydroxide (KOH) etching of silicon nanowires produced by MaCE to open up the cavities and trenches. To the best of our knowledge, this is the first report to use an inkjet of metal nanoparticles on silicon, for the MaCE process. Anisotropic etching of silicon using KOH is well-studied in literature [28]. In our work, we only used a short KOH etching to remove the leftover of silicon nanowires after MaCE. The INKMAC process flow with corresponding scanning electron microscopy (SEM) images is shown in Figure 1.

## 2. Materials and Methods

Printing was done using a FujiFilm Dimatix DMP 2800 (Fujifilm Holdings Corporation, Tokyo, Japan) drop-on-demand printer head with 21 µm diameter nozzles. The silver ink was purchased from Advanced Nano Products (DGP 40LT-15C, Sejong, Korea) with 31.8 wt % of silver nanoparticles and ethylene glycol as the solvent. Printing was done on a single side polished p type boron doped 4 inch silicon wafer of (100) orientation and resistivity of 30–50 ohm·cm. We pre-treated the silicon wafer using 1% HF to remove the native oxide for better adhesion of silver ink to the silicon substrate. We used one nozzle, 23.5 V, 5 kHz and a substrate temperature of 50 °C. The sintering process was needed to remove the polymeric coating around the silver nanoparticles. It took place in an IR oven (infrared IC heater T-962, Puhui Electric Technology CO., Ltd., Shandong, China) at 300 °C for 3, 5 and 7 min to check the effect of sintering time on metallization. 

Chemicals for MaCE, HF (48 wt %) and H_2_O_2_ (30 wt %) were purchased from Sigma Aldrich. Silicon samples with sintered-printed silver ink were cleaved into 1 × 1 cm^2^ pieces and were etched in the H_2_O:H_2_O_2_:HF with two different volumetric ratio solutions of (0:1:1) and (80:55:5) with a fixed total volume of 140 mL at room temperature. Samples were placed face up in the bottom of the etching bath. Etching was followed by careful rinsing in deionized (DI) water and drying with a N_2_ gun. We used 85% purified KOH pellets (Sigma Aldrich, St. Louis, MO, USA) to produce 20 wt % solutions to etch silicon nanowires at 60 °C. Another rinsing and drying step was done after KOH etching as explained.

For optimization of all the steps, 60 samples (1 × 1 cm^2^) were printed and etched. Width of printed lines and diameter of printed dots were investigated using SEM images. Silver nanoparticle size and density were determined by using the public domain Java image processing program ImageJ. After optimization of parameters, 10 identical samples of dots (30 µm in diameter) and 10 identical samples of lines (50 µm of width) were printed and etched to check the process chain reproducibility. 

For the replication process, a silicon template was fabricated using INKMAC. Individual droplets were used with a diameter of 30 µm and center-to-center spacing of 60 µm. Printing was followed by sintering, then 60 min MaCE and 1 min KOH etching as explained. The sample was then coated with a low surface energy self-assembled silane based monolayer (1H, 1H, 2H, 2H-perfluorodecyltrichlorosilane from Sigma Aldrich) for easy replica separation. The gas phase coating was done in a closed Petri dish at 70 °C for 1 h. A polydimethylsiloxane (PDMS) layer (Sylgard 184) with 10:1 mixture of monomers to cross-linking ratio was cured on the template at 70 °C. Simple peel off was used for separation of the replica from the template. Advancing and receding water contact angle of the PDMS replica was measured by an optical goniometer (THETA, Biolin Scientific, Stockholm, Sweden).

For microfluidic channels, first a thin sheet of PDMS was prepared by standard PDMS mixing with a monomer to cross-linker ratio of 10:1. Casting on a polystyrene flat surface and curing in an oven at 70 °C for 2 h was followed consecutively. PDMS-silicon bonding was achieved by 1 min plasma activation (500 sccm of oxygen and 500 W of power).

## 3. Results and Discussion

**Figure 2:**
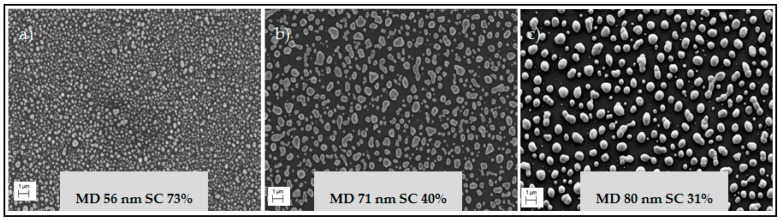
SEM images of printed nanoparticles after, (**a**) 3 min; (**b**) 5 min; (**c**) 7 min infrared (IR) sintering.

As the first step, silver nanoparticles were printed on the HF-treated silicon substrates as either lines or dots (Figure 1a). After printing, the nanoparticles were sintered using an IR oven [29] to remove the polymer coating and to make them conductive to be used as a catalyst. Figure 2a–c shows SEM images of printed silver nanoparticles on silicon after 3, 5 and 7 min sintering with their corresponding mean diameter (MD) and surface coverage (SC) respectively. For a sintering time of 3 min (Figure 2a), the nanoparticle MD is ~56 nm and the SC equals ~73%. The SC of particles decreases while the average MD increases by increasing the sintering time. This happened because of the merging of nanoparticles. We decided to continue the optimization of the process using the 3 min sintering, because higher SC was demanded for MaCE process.

**Figure 3:**
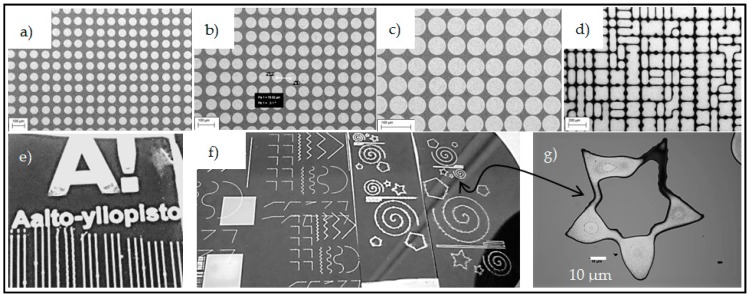
Printed individual droplets with 30 µm diameter and a center-to-center distance of (**a**) 100 µm; (**b**) 80 µm; (**c**) 70 µm and (**d**) Eventually droplets merged. Images of other printed patterns, (**e**) Logo of Aalto University; (**f**) Curves, angles, zigzags and stars; (**g**) Microscopic image of a printed star showing minimum possible feature size.

Inkjet printed lines were used to produce trenches while dots were used to produce cavities. Lines were printed with 2 cm length and widths of 50 µm to 100 µm (Figure 1a). To print dots, individual droplets were used to produce circles with different diameters and pitches. We successfully printed droplets with a minimum diameter of 30 µm. Figure 3a–c shows 30 µm diameter droplets printed and sintered on silicon with a center-to-center distance of 100, 80 and 70 µm, respectively. Droplets merge into each other eventually as pitch is reduced (Figure 3d). Due to inkjet printing flexibility, a variety of patterns have been created, such as curves, zigzags, stars and the logo of Aalto University (Figure 3e–g). The use of commercial silver ink makes the process highly repeatable, as it is a very well-known and established process using the inkjet printing technique and an optimized conductive ink. The silicon wafer is a very good surface for printing. We did not perform any contact-angle measurements with silver ink on the silicon surface, but the surface provides an optimal wettability for precision printing. All the patterns were well defined and showed low deviation (<0.5%) from the designed dimensions.

**Figure 4:**
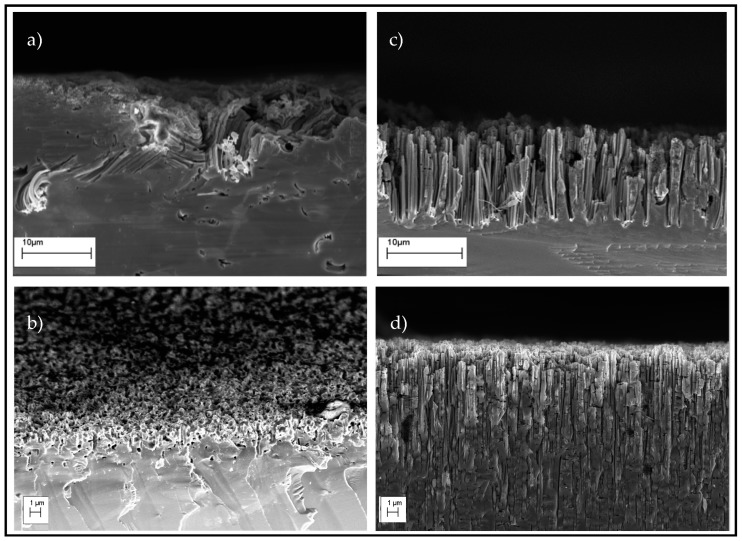
SEM images of the MaCE process of silicon in H_2_O:H_2_O_2_:HF solution, (**a**) 1 min etching in a volumetric ratio of (0:1:1). Etching in (80:55:5) solution for; (**b**) 1 min; (**c**) 10 min; (**d**) 30 min.

The patterns were transferred into the silicon substrate (Figure 4) via wet chemical etching of silicon using silver nanoparticles as catalysts (MaCE) [30]. The optimization of the composition of MaCE solution was done not only in relation with the etching rate but also related to the morphology and the anisotropy of the etched silicon [31]. Two different concentrations of HF and H_2_O_2_ were tested. The solution of H_2_O:H_2_O_2_:HF with a volumetric ratio of (0:1:1) resulted in a high etch rate (10 µm/min), with random movements of metal nanoparticles and consequently random nanostructures (Figure 4a). Lower concentration of HF in MaCE solution (80:55:5) resulted in aligned silicon nanostructures with an etch rate of 1 µm/min. Figure 4b–d shows SEM images of silicon nanostructures produced after 1, 10 and 30 min of MaCE in the (80:55:5) solution. The sintering process was also critical since no etching was observed when we tried to etch silicon without sintering. The removal of the polymer coating from the nanoparticles is thus mandatory for catalytic action in MaCE.

**Figure 5:**
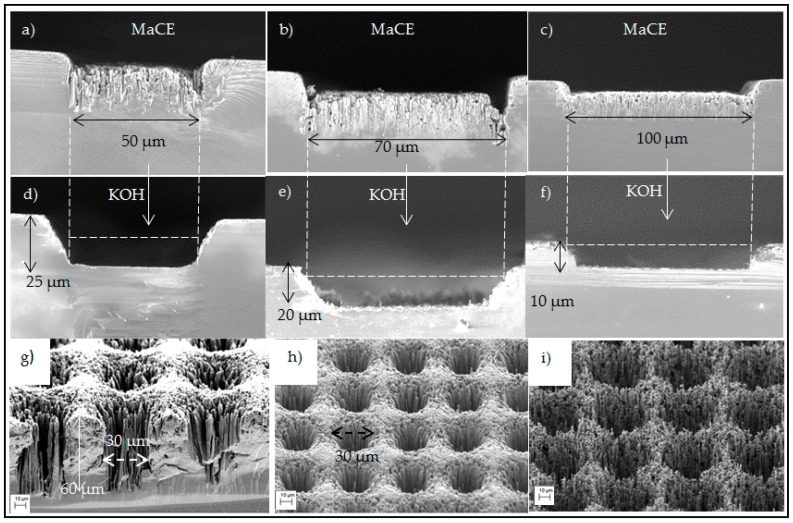
SEM images of printed channels, (**a**–**c**) after MaCE and (**d**–**f**) after KOH etching; (**g**) Cavities after 60 min MaCE and (**h**) after 1 min KOH etching; (**i**) Highly rough surface of the same sample due to increasing the time of KOH etching to 3 min. Dashed lines show the width of printed silver patterns.

Figure 5 shows representative of SEM micrographs of silicon trenches and cavities fabricated by INKMAC. The widths of the trenches ranged from 50 µm to 100 µm and etch depths between 10 µm and 40 µm (Figure 5a–f). The SEM images show a very reproducible process chain. For all the etched samples, the critical dimensions of printed patterns were identical both for cavities and trenches. The maximum aspect ratio of trenches was 0.8 (Figure 1c).

MaCE of 60 min produced 60 µm deep cavities filled with silicon nano-wires (Figure 5g). The diameter of these nanowires was defined by the lateral distance of printed silver nanoparticles, and in our case this was in the range of 100 nm. These high aspect ratio (600:1) silicon nanowires are attracted to each other upon drying to make silicon nano-bushes. The nano-bushes were etched in KOH (Figure 5h). The maximum aspect ratio of etched cavities was 2:1. The minimum size of the final features is limited by the printer resolution which is 30 µm for cavities and 50 µm for trenches. We do not see any practical issue for the maximum feature size limit; it can be in the range of centimeters. The depth of cavities and trenches is very dependent on the adhesion of the silver nanoparticles to silicon substrate during the MaCE process [32]. For better silver–silicon adhesion, we pre-treated the silicon substrate using 1% diluted HF to remove the native silicon oxide [33] in addition to high temperature IR-sintering [34]. We noticed that in MaCE, the etch rate was slowing down after 60 min, most probably due to the delamination of nanoparticles. In fact, we did not detect any further noticeable etching for longer MaCE processes. The final part of INKMAC is the maskless wet etching step that etches away the silicon nanowires to produce fully open silicon trenches and cavities (Figure 1c). We noticed that the timing of this step was crucial. A short etch of 1 min in 60 °C KOH was sufficient to remove the nanowires without excessive surface roughening, while longer etching times led to very rough surfaces (Figure 5i). Although we used a 20 wt % KOH solution, a lower concentration of 1–2 wt % could be enough to remove such nanowires.

**Figure 6:**
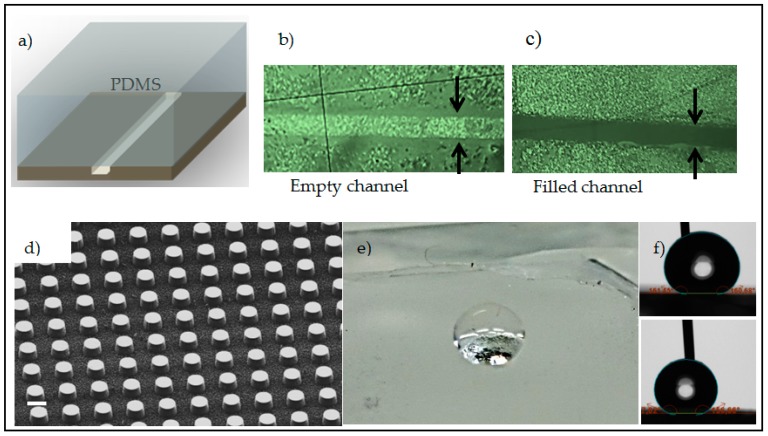
PDMS lid fluidic channel and replica, (**a**) Schematic of the silicon channel lid with PDMS; (**b**) Microscope image of the 50 µm empty channel; (**c**) Microscope image of the same channel filled with colored water without leakage; (**d**) SEM image of a PDMS replica from the silicon master (Figure 5h) scale bar is 30 µm; (**e**) Photo of a droplet on the same replica and (**f**) Contact angle measurement shows advancing 161° (**up**) and receding 158° (**down**).

We demonstrate the suitability of the INKMAC for prototyping of microfluidic structures. An enclosed microfluidic channel was fabricated by bonding a polydimethylsiloxane (PDMS) lid on top of the etched silicon channel (Figure 6a). For the fluidics test, a syringe pump via polytetrafluoroethylene (PTFE) tubes was plugged into an inlet hole punched through the PDMS lid. The channel dimensions were 2 cm length, 50 µm width and 25 µm depth. A flow of colored water was used for increased visual contrast (Figure 6b,c). A volumetric flow rate of 10–100 µL/min was pumped through the channel by a syringe pump and the chip was monitored under a microscope. Within a wide range of flow rates and pressure, bonding demonstrated leak tight performance, indicative that silicon surface quality is not compromised by our process. Noticeably, this was only true for the optimized KOH etching time. Samples etched for longer than 1 min KOH did not provide a leak-tight sealing. We relate this to excessive surface roughness of the silicon.

Further, we demonstrate a replication process using the structured silicon with cavities as a template (Figure 5h) to produce superhydrophobic PDMS. The standard Sylgard 184 PDMS process with a 10:1 ratio was used. The replica shows very high water contact angles (advancing 161° and receding 158°) for a 10 µL droplet with very low roll off angle (<10°) (Video S1). Figure 6d shows the SEM image of the PDMS replica (coated with a 10 nm sputtered gold layer to reduce charging). The height of the pillars was less than the depth of the cavities on the template. This can be either due to uncomplete filling of the master by uncured PDMS or due to failure during the peeling process. A photo of a water droplet in a Cassie state and contact angle measurements for the replica are shown in Figure 6e,f. Unlike the replication of micro-size cavities, the replication of MaCE silicon nanostructures into PDMS was not successful. The high density of silicon nanowires made the peeling process not feasible. To address this issue, a sacrificial release process can be considered as we have reported elsewhere [35], but is not our concern in this report.

## 4. Conclusions

A cheap and easy to use non-lithographic and vacuum-free silicon micromachining is highly desirable both in industrial and academic labs for a variety of applications, such as MEMS devices, energy harvesting systems, solar cells, microfluidic systems and others. INKMAC introduced here is a hybrid technology that uses inkjet printing for patterning and chemical etching to transfer the pattern in silicon substrate. In this work, we demonstrate resolution down to 30 µm diameters of cavities with an aspect ratio of two and 50 µm channels widths with an aspect ratio close to one. These dimensions are sufficient for many of the above-mentioned applications. Further investigation is needed for higher resolution, for example, by using ultra high resolution inkjet systems [36]. Additionally, more study of the MaCE process is needed to provide direct silicon micromachining instead of using KOH for final polishing. 

Although both MaCE and KOH chemical silicon micromachining reported in literature provide more accurate etching with higher aspect ratio structures compared to INKMAC, they are expensive and time consuming. Typically, lithographical methods are needed to define patterns on the surfaces. Patterning is usually followed by a complex vacuum deposition for metallization.

A successful demonstration of pressure-driven flow indicates that our technology is suitable for microfluidics. Hybrid systems with silicon channels and a transparent PDMS lid are useful in many applications where, for instance, heated channels are needed while optical detection is used. Compared to other non-lithographic methods, such as 3D printing, INKMAC has much better resolution and enables high aspect ratio structures. We also produced superhydrophobic polymers by using the structured silicon as a template for PDMS replication. The PDMS replica showed a water contact angle of 160° with a rolling angle of <10° without any further surface modification. The collapsed silicon nanowire bundles which were produced during the drying step after MaCE can also be utilized for surface engineering applications, such as hydrophobic/hydrophilic patterning [37,38] or superhydrophobic surfaces [35,39]. 

## Supplementary Materials

The following are available online at www.mdpi.com/2072-666X/7/12/222/s1, Video S1: Superhydrophobic PDMS replica.
